# Information processing via physical soft body

**DOI:** 10.1038/srep10487

**Published:** 2015-05-27

**Authors:** Kohei Nakajima, Helmut Hauser, Tao Li, Rolf Pfeifer

**Affiliations:** 1 The Hakubi Center for Advanced Research & Graduate School of Informatics, Kyoto University, 606-8501 Kyoto, Japan; 2 Department of Engineering Mathematics, University of Bristol, Bristol BS8 1UB, United Kingdom; 3 Department of Engineering and Information Technology, Bern University of Applied Sciences, 2501 Biel, Switzerland; 4 Institute for Academic Initiatives, Osaka University, Osaka 560-8531, Japan; 5 Department of Automation, Shanghai Jiao Tong University, Shanghai 200240, China

## Abstract

Soft machines have recently gained prominence due to their inherent softness and the resulting safety and resilience in applications. However, these machines also have disadvantages, as they respond with complex body dynamics when stimulated. These dynamics exhibit a variety of properties, including nonlinearity, memory, and potentially infinitely many degrees of freedom, which are often difficult to control. Here, we demonstrate that these seemingly undesirable properties can in fact be assets that can be exploited for real-time computation. Using body dynamics generated from a soft silicone arm, we show that they can be employed to emulate desired nonlinear dynamical systems. First, by using benchmark tasks, we demonstrate that the nonlinearity and memory within the body dynamics can increase the computational performance. Second, we characterize our system’s computational capability by comparing its task performance with a standard machine learning technique and identify its range of validity and limitation. Our results suggest that soft bodies are not only impressive in their deformability and flexibility but can also be potentially used as computational resources on top and for free.

Recently, soft machines have attracted attention in a number of research fields. Their soft bodies can undergo dramatic changes in their morphologies to adapt to various environments^1^,^2^. They deliver vital applications for carrying fragile objects, for human-robot interaction, and for search and rescue in emergency situations, mostly due to their inherent softness that results in increased adaptivity and less damage during contact^3^,^4^. Furthermore, their production costs are relatively low, so they can be easily incorporated into a wide range of machines for various purposes^5^.

Concomitant to these benefits is a great challenge in controlling their body dynamics. Generally, soft body dynamics exhibit a variety of properties, including nonlinearity, memory, and potentially infinitely many degrees of freedom^1^,^4^. In addition, their degrees of freedom are often larger than the number of actuators (i.e., underactuated systems), which leads to well-known difficulties in controlling them^6^.

Here, we demonstrate that these seemingly undesired properties can in fact be highly beneficial in that they allow us to use soft bodies as computational resources. Our approach is based on a machine learning technique called *reservoir computing*, which has a particular focus on real-time computing with time-varying inputs and is suited to emulate complex temporal computations^7^,^8^,^9^,^10^,^11^. Its framework has been proposed to overcome a limitation in the conventional attractor-based neural networks that takes a certain amount of time to converge to an attractor state, which is less suitable for real-time computing. Our approach places more emphasis on the transient dynamics of high-dimensional dynamical systems^9^,^10^,^11^,^12^, which are typically referred to as the *reservoir*. To have computational capabilities, a reservoir should have the properties of input separability and fading memory^9^. Input separability is usually achieved by nonlinear mapping of the low-dimensional input to a high-dimensional state space, similar to the function of a kernel in machine learning^13^. Fading memory is a property of a system that retains the influence of a recent input sequence within the system, which permits the integration of stimulus information over time. This enables reproducible computation for which the recent history of the signal is significant. If the dynamics of the reservoir involve enough nonlinearity and memory, emulating complex, nonlinear dynamical systems only requires adding a linear, static readout from the high-dimensional state space of the reservoir. A number of different implementations for reservoirs have been proposed, such as abstract dynamical systems for echo state networks^7^,^8^ or models of neurons for liquid state machines^9^, the surface of water in a laminar state^14^, and nonlinear mass spring systems^15^,^16^,^17^,^18^,^19^. Lately, electronic and optical implementations have also been reported^20^,^21^,^22^,^23^. These examples clearly illustrate that the prerequisites to be a reservoir do not depend on the specific implementation of the system but rather on the properties of the system (i.e., input separability and fading memory).

In this study, we use a simple but powerful physical platform with a soft silicone arm to demonstrate that soft body dynamics can be used as a reservoir. Previously, we showed that the body dynamics generated by this soft silicone arm can be used to emulate functions that require short-term memory and embed robust closed-loop control into the arm on the basis of a Boolean function^24^. In this study, we extend our previous approaches and aim to emulate nonlinear dynamical systems based on continuous functions, which are more frequently used in real-world applications. For example, controllers, which are often defined as continuous dynamical systems, are particularly interesting target systems for such an emulation^25^. This would imply that we can use the physical body of a soft machine to carry out computations needed to calculate complex time-dependent control actions and embed motor programs into the body through feedback loops. By comparing with standard machine learning techniques, we systematically characterize the information processing capacity of the soft body dynamics and show that the soft body dynamics can be exploited as a part of computational device.

## Results

### Information processing scheme via a soft silicone arm

**Figure 1:**
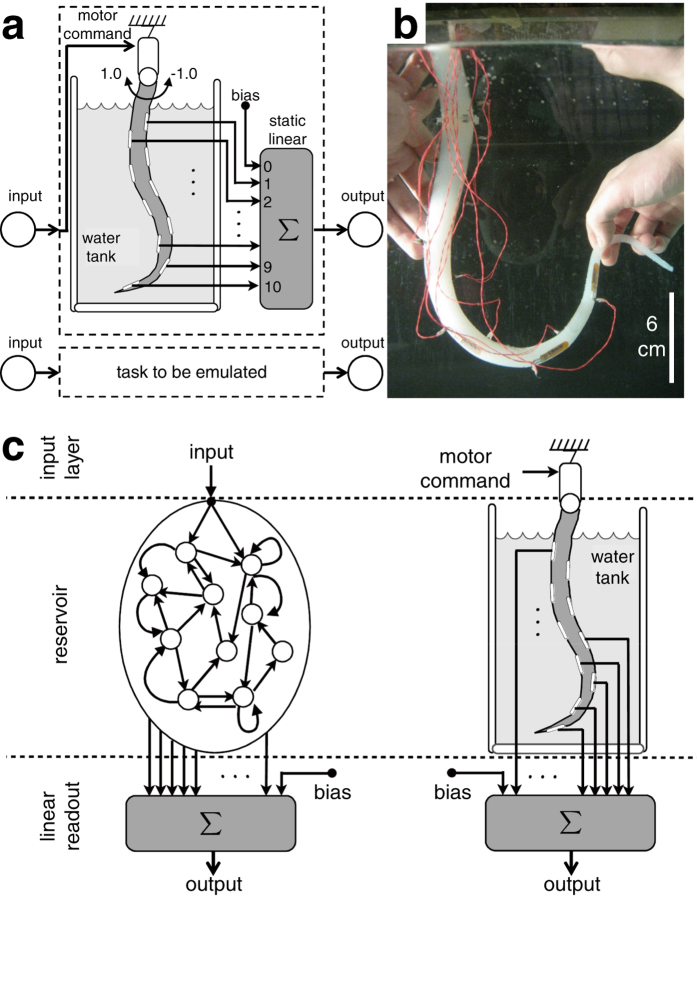
Platform setup for a soft silicone arm and schematics showing the information processing scheme using the arm. (**a**) Schematics showing a scheme using the soft silicone arm as a part of a computational device. The arm embeds 10 bend sensors and is immersed underwater. Inputs are motor commands that generate arm motions, and the embedded bend sensors reflect the arm posture for each timestep. Corresponding system outputs are generated by the weighted sum of the sensory values. (**b**) Picture showing the soft silicone arm used in this study. (**c**) Schematics expressing an analogy between a conventional reservoir computing system and our system. In a conventional reservoir system, randomly coupled abstract computational units are used for the reservoir, whereas our system exploits a physical reservoir whose units are sensors that are coupled through a soft silicone material. Our question here is whether our physical reservoir can perform tasks of nonlinear dynamical systems emulations, which are often targeted with conventional reservoir computing systems and are useful in the context of control.

The platform consists of a soft silicone arm, its sensing and actuation systems, data processing via a PC, and a water tank containing fresh water as an underwater environment (Fig. 1(a,b), and Supplementary Fig. S1(a)). The soft silicone arm used in this study is the one developed in^24^, and it has similar material characteristics to the one proposed in^26^, which was inspired by the octopus (Fig. 1(b). A comparable structure has been commonly used for soft robotic arms (see, e.g., [27,28]). Throughout this study, the behavior of the system is observed from one side of the tank, and we use terminologies, such as “left” or “right,” with respect to this fixed point of view. The arm has 10 embedded bend sensors within the silicone material. A bend sensor gives a base value, which is set to 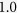, when it is straight. If it bends in the ventral side, the sensor value is lower than 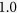, and if it bends in the dorsal side, the value is larger than 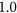; the change in value reflects the degree of bend in each case (see Methods section and^24^ for detailed information). The sensors are embedded near the surface of the arm with their ventral sides directed outward and are numbered from the base toward the tip as 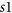 through 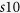. They are embedded alternately, with the odd-numbered sensors (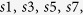 and 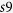) on the right side of the arm and the even-numbered sensors (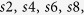 and 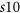) on the left Fig. 1(a). The base of the arm can rotate left and right through the actuation of a servo motor. The motor commands 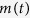 sent from the PC control the position of the base rotation and are real values in the range of 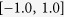, where 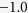 and 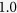 correspond to the maximum right position or the maximum left position, respectively (Fig. 1(a) and Supplementary Fig. S1(b). The actual servo motor positions are also sent to the PC to monitor the current position of the base rotation 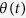, which is also in the range of 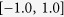. Our system has only one active degree of freedom but a high number of passive degrees of freedom in the silicone arm. Hence, we deal with an underactuated system^6^. Throughout this study, the unit of timestep 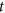 corresponds to the sensing and actuation loop of the PC (this is about 0.03 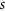 in physical time). Further details on the platform setup are given in Methods section.

By generating passive body dynamics resulting from the interaction between the water and the soft silicone material^29^,^30^, we will show that the sensory time series reflected in the body dynamics can be used to emulate the desired nonlinear dynamical systems, which are often targeted with a recurrent neural network learning or reservoir computing approach Fig. 1(c). For this purpose, we first need to define how to provide inputs 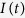 to the system and how to generate corresponding outputs 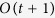. In this study, we apply the motor command as an input, and the output is generated by a weighted sum of the 10 sensory values and a constant valued bias set to 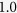 Fig. 1(a). The output is defined as follows:
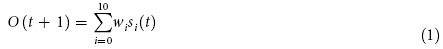
where 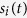 (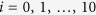) denotes a sensory value of sensor number 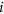 at timestep 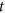, where 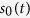 is a bias, and 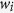 is a corresponding linear readout weight. To emulate the desired nonlinear dynamical systems with our system, we adjust only these linear readout weights 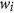, which are fixed after learning. By collecting 10 sensory values and a bias for 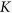 timesteps, we can generate an 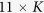 matrix 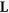 as the training dataset. In addition, we collect the corresponding target outputs over time for 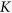 timesteps in a matrix 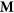. Then, the optimal linear readout weights, 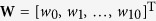, can be obtained by **W=L**^†^**M**, where **L**^†^ is the Moore-Penrose pseudo-inverse, because **L** is not a square matrix in general. To emulate nonlinear dynamical systems successfully with this setup, the required memory and nonlinearity for such emulations will have to be provided through the properties of the soft silicone arm and from the resulting dynamics because we are only adding a static and linear readout.

To evaluate the computational power of our system, we use the emulation tasks of nonlinear dynamical systems, called *nonlinear auto-regressive moving average* (NARMA) systems, which are standard benchmark tasks in the context of machine learning (e.g., recurrent neural network learning). This task presents a challenging problem for any computational system because of its nonlinearity and dependence on long time lags^31^. The first NARMA system is the following second-order nonlinear dynamical system:
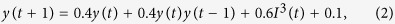
where *y*(*t*) denotes the output of the system. This system was introduced in^32^ and used, for example, in^15^,^17^. For descriptive purposes, we call this system NARMA2. The second NARMA system is the following nonlinear dynamical system that has an order of *n*:
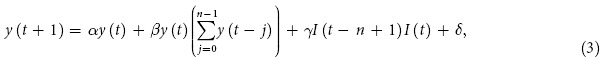
where (*α*,*β*,*γ*,*δ*) is set to (0.3,0.05,1.5,0.1). Here, *n* is varied for values of 5,10,15, and 20, and the corresponding systems are called NARMA5, NARMA10, NARMA15, and NARMA20, respectively. In particular, NARMA10 with this parameter setting was introduced in^32^ and broadly used (see, e.g., [11,15,17]).

The input *I*(*t*) is expressed as a product of three sinusoidal functions with different frequencies:
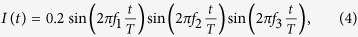

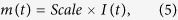
where (*f*_1_,*f*_2_,*f*_3_) is set to (2.11,3.73,4.33), and the parameter *T* controls the phase velocity of the input time series. As *T* increases, the input time series becomes slower in varying. In our experiments, we set *T* to 100,150,200,250,300,350, and 400. We have heuristically found that when *T* becomes smaller than 100, the motor tends to overheat and stop, whereas if *T* is larger than 400, the sensory values become too small because of the small degree of bending in the arm (Supplementary Fig. S2(a) and (b)). The parameter *Scale* linearly scales the input *I*(*t*) to the range [−1.0,1.0] and is set to 5.0. As for the input to the NARMA systems, randomly drawn real values are common. However, as explained earlier, unlike conventional computational units, such as artificial neural networks, the inputs to our system are the motor commands, which transform the input values to the mechanical realm. Thus, a drastic and frequent transition of the motor command can result in motor overheat and a complete stop. For this reason, we exploited a slow varying time series for the input; this approach has also been frequently applied to evaluate system performance (see, e.g., [15,17]). According to the sent input, the system should simultaneously emulate the aforementioned five nonlinear dynamical systems (NARMA2, NARMA5, NARMA10, NARMA15, and NARMA20), which we call multitasking.

For the experimental procedure, the soft silicone arm is first set to the resting state (*θ*(*t*) = 0), and before beginning the experiment, we run the arm with input as defined in equation (4) for *t*_*ini*_ timesteps. This phase aims to set the different initial positions of the arm for each experimental trial; *t*_*ini*_ is randomly determined from 0 to 10000 timesteps for each trial. The actual experimental trial consists of 11000 timesteps, where the first 1000 timesteps are for the washout, the following 5000 timesteps are for the training phase, and the final 5000 timesteps are for the evaluation phase. The length of the training and evaluation phases is set to characterize the generalization capability of the system effectively, where the input and target output time series in the evaluation phase will not appear in the training phase (note that our input signal has a period of 100 × *T* timesteps). After *t*_*ini*_ timesteps, we continue running the arm with equation (4) and the actual experiment begins. By collecting the sensory time series and the corresponding target outputs for each task in the training phase, we train the linear readouts for five outputs, which correspond to the five target NARMA systems, by using the previously explained procedure. For the evaluation phase, the trained linear readouts are used to generate system outputs.

To characterize the contribution of the soft silicone arm dynamics explicitly, we have compared the task performance with a simple linear regression (LR) model, *O*(*t* + 1) = *w*'_1 _× *I*(*t*) + *w*'_0_, where *w*'_0_ and *w*'_1_ are trained using the same time series as in the training phase. Note that this corresponds to the case in which no physical body is available, and only the raw input remains for LR. From this comparison, for any system performance better than that of this model, we can conclude that the soft body dynamics has contributed to the emulation task.

We evaluate the performance of the system output in the evaluation phase by calculating the normalized mean squared error (NMSE) with the target output:
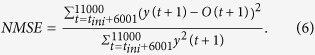
For descriptive purposes, we denote NMSE_*system*_ for the NMSE of our system and NMSE_*LR*_ for that of the LR model.

### Emulation of nonlinear dynamical systems

**Figure 2:**
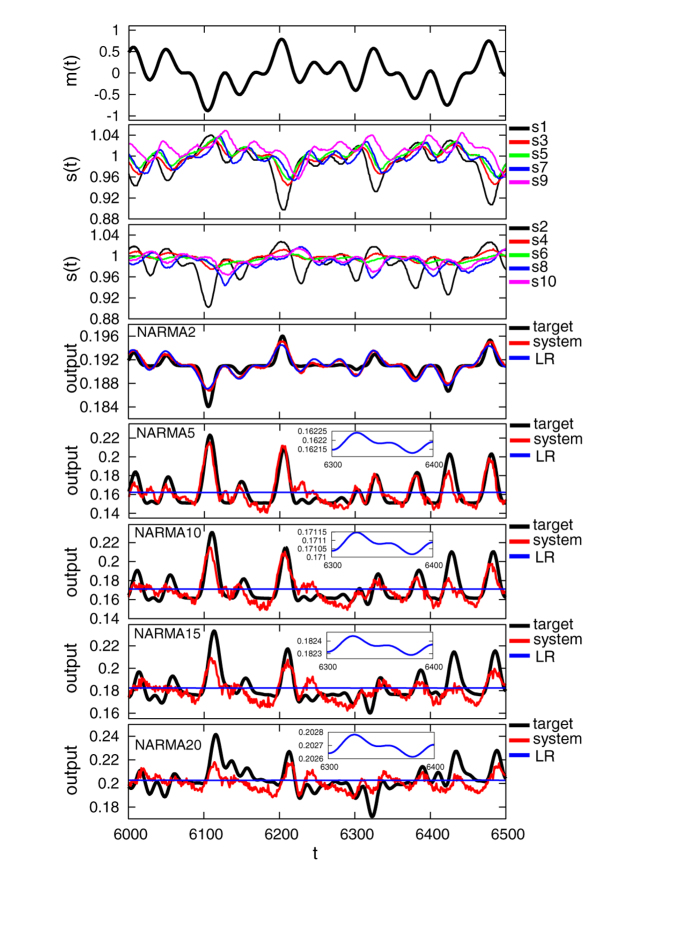
A typical example of the task performance in terms of time series when *T* = 400 in the evaluation phase. From the upper to the lower plots, the time series of the motor command, the corresponding sensory values (odd-numbered sensors and even-numbered sensors), and the outputs for NARMA2, NARMA5, NARMA10, NARMA15, and NARMA20 are depicted. For each plot of the output, the time series for the system output as well as the target output and the output for the LR model is overlaid for comparison. Note that the output of the LR model, especially in NARMA5, NARMA10, NARMA15, and NARMA20, is not a constant but a scaled version of the input with an offset (see the inset that scales up the output for the LR model from timestep 6300 to 6400 in each plot).

**Figure 3:**
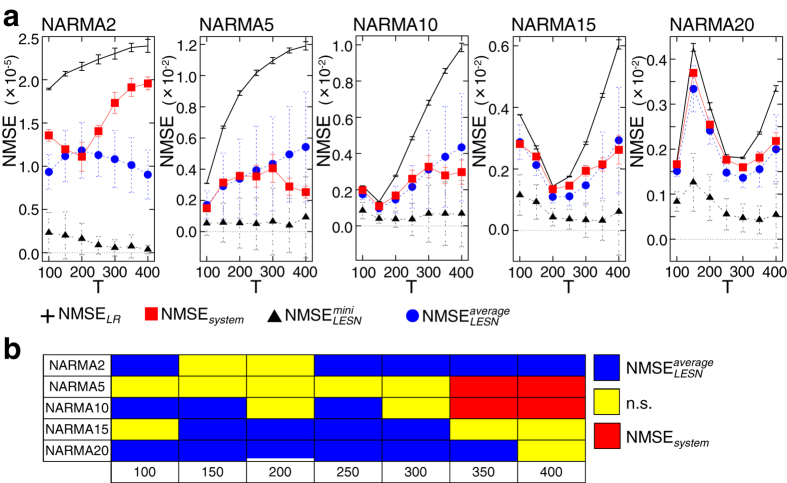
Comparisons among the average NMSE_*system*_, NMSE_*LR*_, 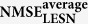, and for all NARMA tasks for each setting of *T* (a) and a diagram summarizing the significant differences between NMSE_*system*_ and 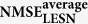 (b). In (**a**), error bars represent standard deviations. For each task, the average NMSE_*system*_ is significantly lower than the average NMSE_*LR*_ (seemingly overlapping plots in, e.g., the NARMA10 task with *T* = 100 and 150, the NARMA15 task with *T* = 200, and the NARMA20 task with *T* = 100 and 250, are due to the scaling of the figures), while the average 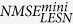 is significantly lower than the average NMSE_*system*_ for each setting of *T* (Supplementary Table S1). In (**b**), among NMSE_*system*_ and 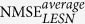, the significantly lower one with *p* < 0.05 is depicted for each experimental condition. Note that “n.s.” represents “not significant.” All the information, including the average NMSEs as well as the results for significant tests in each experimental condition, is given in Supplementary Table S1.

A typical example of the input and sensory time series and the performance for each NARMA task during the evaluation phase is shown in Fig. 2. In the figure, the parameter *T* of the input is set to 400, and the performance of the LR model is also presented for comparison. We can see that our system output shows better performance than the LR model in all presented NARMA tasks. For the emulation task of the NARMA2 system, the LR model also shows relatively good performance because less memory is required to emulate the system. (Note that NMSE_*system*_ and NMSE_*LR*_ for NARMA2 in the trial presented in Fig. 2 are 1.8 × 10^−5^ and 2.3 × 10^−5^, respectively.) However, not surprisingly, in NARMA tasks that have larger degrees of order, the LR model calculated on the raw input stream clearly fails to emulate the target dynamical systems. This result implies that the required temporal integration and nonlinearity are contributed by the soft body dynamics. We can also observe in Fig. 2 that the relatively successful performance of our system gradually decreases as the degree of memory of the task increases, suggesting the limitation of the memory capacity of the soft body dynamics that can be exploited. Similar tendencies have been observed in each setting of *T* for the input stream. This result points to the physical constraints of the platform used. Examples of the system performance and the corresponding arm motion, as well as the sensory time series during the evaluation phase for *T* = 100,200,300, and 400, can be found in Supplementary Video S1. We have calculated the average NMSE for each NARMA task by iterating the experiment for 20 trials for each input setting of *T* (Fig. 3(a)). As shown in Fig. 3(a), for all NARMA tasks at each *T* setting, NMSE_*system*_ shows a lower value than NMSE_*LR*_. The statistical comparisons show a significant difference with *p* < 0.01 for all the conditions (Supplementary Table S1), a result confirming that our system successfully exploits the nonlinearity and memory originating from the passive body dynamics of the soft silicone arm to perform the tasks.

### Comparing the system performance with that using a standard reservoir system

To further characterize the computational power of our system, we have compared our system performance with that of conventional reservoir system called a leaky integrator echo state network (LESN), introduced in^33^. Like other reservoir systems, LESN has input and output layers and a reservoir, whose computational unit is equipped with a leaky integrator. Because we use a superimposed sine wave for the input time series, the NARMA systems to be emulated here tend to have slow dynamics, as shown in Fig. 2. LESN is better suited for these slow dynamics than the conventional ESN because the reservoir unit has individual state dynamics^33^. This is the reason for the choice of LESN for our comparisons (similar discussions can be found in^34^,^35^,^36^). For effective comparisons with our system, we investigate the performance of LESN with the same number of computational nodes (10 fully coupled reservoir nodes with one bias term), the same input time series, and the same training and evaluation data sets.

It has been reported in^33^ that the optimal reservoir of LESN depends mainly on two parameters, the leaky rate *a* of a unit and the reservoir weight matrix spectral radius *ρ*, where the effective range of the parameters is investigated as 0 < *ρ* ≤ *a* ≤ 1. (Note that in our experiment, when training the readout for LESN, we added a small amount of noise to the reservoir states in the range of [−*ν*,*ν*] in the training phase. The setting of *ν* was investigated in Supplementary Fig. S3 in detail and is here set to *ν* = 10^–9^.) We have varied these parameters exhaustively for all values of 0 < *ρ* ≤ *a* ≤ 1 in increments of 0.01 and investigated the performance of LESN for each parameter setting. In comparison with our system, we have focused on two factors: (I) the global average of NMSE and (II) the global minimum of NMSE (with precision to the second decimal point) across the entire parameter space of (*a*,*ρ*). Intuitively, (I) represents the performance of an arbitrary prepared LESN, and (II) represents that of an optimal LESN. For the analysis, we prepared 50 randomly initialized LESNs, and for each (*a*,*ρ*), we calculated the average NMSE using the same 20 trials, input and target output data sets for each LESN. For the case of (I), across the entire (*a*,*ρ*) space, we collected the average NMSEs that have lower error than the average NMSE_*LR*_ and averaged them. We call this value 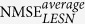
For the case of (II), we collected only the minimum value of the average NMSEs across the entire (*a*,*ρ*) space and call this 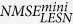. Finally, the 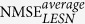 and the 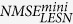 are averaged over 50 LESNs, which have been used for the comparison. Further details on the LESN settings and the error comparison procedures are provided in Methods section.

The results for the average 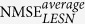 and average 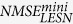 are plotted in Fig. 3(a). At first, we can see that in all the NARMA tasks, 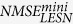 shows a lower value than NMSE_*system*_ for every setting of *T*. This is further confirmed via significant tests, in which all the comparisons between 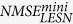
and NMSE_*system*_ show a significant difference with *p* < 0.01 (Supplementary Table S1). This result implies that the task performance of LESN with optimal reservoirs (i.e., case II) is significantly better than that of our system. By contrast, for the comparisons with 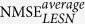 (i.e., case I), we can see that for some settings of *T* in each NARMA task, NMSE_*system*_ shows values lower than or similar to 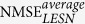 (Fig. 3(a)). The statistical comparisons reveal that NMSE_*system*_ is significantly lower than 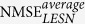 for NARMA5 and NARMA10 tasks with *T* = 350 and 400, and simply show no significant difference in several experimental conditions (Fig. 3(b) and Supplementary Table S1). Furthermore, we analyzed the averaged ratio of the parameter settings (*a*,*ρ*) that have lower values than NMSE_*system*_ against the entire parameter space and found that in some experimental conditions, the averaged ratio is less than 0.5 for each task, suggesting that more than half of the LESNs perform worse than our system (Supplementary Fig. S4 (a) and (b)). These results imply that in some experimental conditions, the task performance of our system can be better than that of LESN or exhibit performance similar to that of LESN, whose reservoir is arbitrarily chosen in a statistical sense.

Not surprisingly, the performance of our physical system was generally not as good as that of the optimal LESN. Nevertheless, on average, our proposed setup performed comparable to the LESN approach. We here must take into account that LESNs represent a machine learning technique, which is designed exactly for these kinds of tasks, whereas the primary function of a soft body is not the emulation of dynamical systems. We merely exploit the given complex dynamic properties of the “softness” of the body (i.e., nonlinearity and memory) for our purposes. This implies that soft bodies have multifaceted usages and advantages, which are not only appealing in their deformability and flexibility but can also potentially be exploited as computational resources.

## Discussion

In this study, we have systematically demonstrated that body dynamics originating from a soft silicone arm can be exploited to emulate nonlinear dynamical systems. We confirmed this by comparing the task performance with that of the LR model, from which we found that in all our tasks and for any settings of *T* for the input, our system outperformed the LR model. Hence, the body dynamics contributed positively to all computational tasks. Our scheme enabled us to emulate multiple nonlinear dynamical systems simultaneously by using the dynamics generated from a single soft body. We further characterized the performance of our system by comparing it with that of LESN. As expected, our physical system performed worse than an artificially optimized LESN. However, our setup had a performance comparable to or even better than that of LESN, whose parameters (*a*,*ρ*) were chosen randomly under various experimental conditions. Our results suggest that the incorporation of soft materials into the body of machines or robots also represents the addition of potential computational resources that can be exploited. If the appropriate conditions for the actuations are set, and the appropriate computational tasks are defined, the soft body can even be successfully exploited as a part of a computational device. Considering that the body has primary functions other than computation, we could even argue that this feature comes along with soft bodies for free.

To improve the computational power of the physical reservoir, different types of sensing systems, actuations, material properties, and body morphologies can be explored. For example, a number of materials that show interesting functionalities with respect to our presented setup have been proposed (see, e.g., [37,38]). These materials can be potentially used for the design of the physical bodies of soft machines. In particular, as explained earlier, the key properties that a mechanical reservoir should have in our scheme are input separability, which is achieved by nonlinearity and high-dimensionality of the reservoir, and fading memory. In our system, these properties were naturally demonstrated in the body dynamics through the interaction between the water and the material and morphological structure of the soft silicone arm. Input separability was achieved by the nonlinear mapping of the actuation signal, which is due to the interaction between the water friction and the cone-shaped deformable morphology of the arm, into the higher dimension of the soft body. The slow relaxation of the body dynamics against the actuation, which is due to the damping effect provided by the underwater environment, implemented the fading memory property. This observation emphasizes that the dynamic interaction between the controller, the body, and the environment is another important factor to be considered and explored to improve the computational power of the system. Moreover, because the prerequisites to be a reservoir do not depend on the specific implementation but rather on the properties of the system, it would be possible to use modular systems or multi-agent systems (e.g., [39]) as physical reservoirs as this would enable the reconfiguration of the entire system as well as the alteration of the computational power of the system dynamically in real-time.

Our presented technique can be applied in a number of ways. For example, it can be used to produce nonlinear limit cycles, which enable locomotion in robots (e.g., [40]). It can be embedded into the body in a closed-loop manner without the additional support of nonlinearity and memory from an external controller. Recently, this line of study has been started with the use of physical platforms (e.g., [24,41,42]). Furthermore, with a novel technique to implement linear readouts as a device, it would be possible to distribute desired controls to spatially distant points of the body through the usage of body dynamics only by actuating local body parts. This approach would be especially useful when the actuation points are limited because of physical constraints of the platform. Including these, we expect our approach not only to open up various engineering applications for the use of soft materials but also to inspire further experiments with novel concepts for computing in multiple fields.

## Methods

### Platform setup

The overall experimental platform, which is shown in Supplementary Fig. S1(a), consists of a soft silicone arm, its actuation, sensing, and control systems, as well as a water tank containing fresh water as the working environment. The water tank is 100 cm long, 50 cm wide, and 50 cm deep. For each experimental trial, the amount of water in the tank is controlled, so that it has the same height as the apical surface of the plastic material of the base when the arm is aligned vertically to the water surface. The soft arm is made of silicone rubber (ECOFLEX 00-30 from Smooth-On, Inc.) in a cone shape that is 44.7 cm long with a radius of 1.4 cm at one end (base) and a radius of 0.15 cm at the other end (tip). This setting ensures that the arm will not touch the ground and walls during movement. During experiments, the arm is immersed in the water and actuated by a servo motor (Dynamixel RX-64) at the arm base, which consists of rigid plastic and is directly connected to the motor (Supplementary Fig. S1(b)). The servo motor is fixed on a plexiglass plate that is placed on top of the water tank (Supplementary Fig. S1(a)). The maximum right position (*θ*(*t*) = −1.0) and the maximum left position (*θ*(*t*) = 1.0) of the motor rotation, which are about 46.4 degrees by setting the origin of the rotation angle (0 degrees) when the arm is aligned vertically to the water surface, were heuristically determined to avoid damaging the motor components (Supplementary Fig. S1(b)). Ten sensors embedded near the surface in the arm are used to detect the amount of bending of the arm during experiments. The flexible, lightweight bend sensor (Flexpoint Sensor Systems, Inc.) is roughly 3.2 cm long, including connectors, 0.7 cm wide, and less than 0.1 cm thick^43^. It contains a layer of coated bend-sensitive ink, which converts mechanical deformation into electric resistance. Typical sensory response curves can be found in the design manual of the provider^44^. The sensors are connected to a sensory board by red wires. Using L-shaped wire connectors and placing the electrical wires completely outside of the arm, we can easily replace the wires and make them free from bending stress. The wires are set as carefully as possible so as not to affect arm motion. Finally, a Java program running in the PC reads and records the sensory data, as well as the motor commands at each timestep. Further details on the sensory board setup and the manufacturing process of the arm can be found in^24^.

### Specifications of the motor commands and the corresponding sensory responses

As explained in the main text, we used the position control of the servo motor according to the motor command *m*(*t*) sent from the PC. This setting inevitably generates a slight difference between the motor command and the actual position of the base rotation *θ*(*t*) for each timestep because the motor rotates in a finite velocity. Because this physical constraint is difficult to avoid, we include this as our experimental condition and provide its specifications. As is given in equation (4) in the main text, we used a super-imposed sine wave as the input time series with a parameter *T* controlling the phase velocity for our experiments. We investigated the normalized mean squared error between *m*(*t*) and *θ*(*t*) (NMSE_*motor*_) during the experimental trial, which is defined as
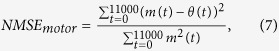
where NMSE_*motor*_ is averaged over 20 trials for each *T*. Supplementary Figure S2(a) plots the results of the averaged NMSE_*motor*_, where the value of the averaged NMSE_*motor*_ decreases as *T* increases. This result is understandable because when *T* increases, the transition velocity of the input slows down, and the motor can easily follow the sent motor command. Note, however, that in principle, NMSE_*motor*_ will not reach 0 value even if *T* increases more. Furthermore, as *T* increases, the amplitude of sensory responses decreases because the arm will not largely bend as a result of the slowly varying motor command. We investigated this by checking the variance of each sensory value in each experimental trial. The averaged variance (20 trials) of each sensory value according to each *T* setting is shown in Supplementary Fig. S2(b). By setting the value of *T* smaller than 100, we frequently observed a total stop of the motor due to overheating. If *T* becomes larger than 400, the sensory responses become smaller as we saw here, so monitoring the arm’s body dynamics would be difficult. Thus, in our experiment, we restricted the range of *T* from 100 to 400. Noteworthy, this setting is dependent on the multiple factors of our platform, such as the morphology and softness of the arm, the effect of the water friction on it, the type of motor that we used, and so on.

### LESN settings

To characterize the computational power of our system in the main text, we compared the task performance of our system with that of LESN^33^. LESN is a type of recurrent neural network that has *N*_*net*_ reservoir units, which are leaky integrators, *N*_*in*_ input units, and *N*_*out*_ output units. Throughout the analysis, *N*_*net*_ is set to 10, which is the same number of sensors in our system, *N*_*in*_ is set to 1, and *N*_*out*_ is set to 5 for five NARMA tasks. Furthermore, to compare the task performance directly, we used the same input and target output data set with our system for the washout (1000 timesteps), the training phase (5000 timesteps), and the evaluation phase (5000 timesteps) for 20 trials for each setting of *T*, as described in the main text. The activation of the *i*th internal unit is *x*_*i*_(*t*) (*i* = 1,...,*N*_*net*_), and the activation of the input and output units are *I*(*t*) and *O*(*t*), respectively. The connection weights for the *N*_*net*_ × *N*_*net*_ reservoir network connecting the *i*th unit with the *j*th unit are denoted as *w*_*ij*_, and the connection weights going from the input unit to the *i*th internal unit are denoted as 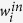. The readout weights 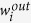 go from *N*_*net*_ reservoir units and 1 bias to the output unit (where 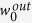 is a bias term), and they are trained in the same procedure as explained in the main text for each experimental trial in each task condition. Although the original LESN is defined as continuous-time dynamics due to the leaky integrator unit, it is shown in^33^ that by using a Euler discretization procedure, it can take a discretized form without loss of generality. We use this discretized version of LESN, which is expressed as
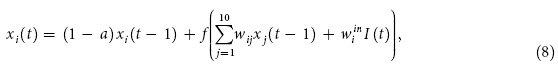

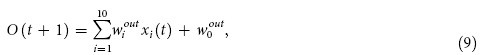
where a > 0 and *f* are the leaky rate of a unit and a tanh function, respectively.

In^33^, the important global parameters to form an optimal reservoir of LESN are discussed, and particularly in our setting, they correspond to (i) the scaling of input weights, (ii) the reservoir weight matrix spectral radius *ρ*, (iii) the leaking rate *a*, and (iv) the noise intensity *ν*, which represents the noise (in the range of [−*ν*,*ν*]) added to the state of the reservoir units during the training phase. For the scaling of input weights 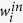, we assigned random real values in the range of [−1.0,1.0], and this setting is fixed throughout our experiments. For the reservoir weight matrix spectral radius *ρ* and the leaking rate *a*, it is investigated in^33^ that their effective range has a relation of 0 < *ρ* ≤ *a* ≤ 1. As explained in the main text, we varied these parameters in increments of 0.01 and analyzed the task performance of LESN. To set the reservoir weight matrix spectral radius, we first assigned random real values in the range of [ − 1.0,1.0] for *w*_*ij*_ and updated the weights to have a unit spectral radius by normalizing the original weights with its spectral radius. Then, the weights are scaled with the required weight matrix spectral radius *ρ*. Finally, for the noise intensity *ν* during the training phase, we found that its value is crucial to the task performance of LESN. For example, when we trained LESN with *ν* = 0 (no noise added) for the given input and target output data, we often observed that the error between the LESN output and the target output showed extremely high values during the evaluation phase. This result is speculated to be caused by over-fitting. However, when we added relatively larger noise, we observed that the task performance of LESN became worse. To determine the relevance of noise intensity to the task performance, we analyzed 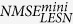, which is defined in the main text, over 20 trials for each task with each setting of *T* by varying *ν* from 10^–1^ to 10^–9^ and by averaging over 30 different LESNs (Supplementary Fig. S3). As shown in Supplementary Fig. S3, when we decrease the noise intensity from 10^−1^, the averaged 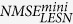 tends to decrease and converges to some value at a certain noise level in all the conditions. Accordingly, for the analyses in the main text, we set an adequately small noise intensity of *ν* = 10^–9^ to guarantee the good performance of LESN, and we fixed it throughout our experiments. The detailed procedures on the statistical comparisons of the task performance of LESN and the LR system with that of our system can be found in the following sections.

### Two criteria for the analyses of LESN

As explained in the main text, to effectively compare the task performance of LESN and that of our system equipped with a soft silicone arm, we set two criteria for the analyses of LESN based on the parameter spaces of *a* and *ρ* (0 < *ρ* ≤ *a* ≤ 1 in increments of 0.01). The first one is to compare with the global average of the performance of LESN in terms of 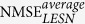, which was defined in the main text. To calculate the 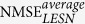, we prepared an LESN as explained in the previous section and in the main text, and then for each parameter set of (*a*,*ρ*) for each NARMA task and the parameter setting of *T*, we calculated the averaged NMSE over 20 experimental trials with the same training and evaluation data set as that used in our system. By excluding NMSEs that have larger values than NMSE_*LR*_ (to avoid extremely large errors caused by the over-fitting for fair comparisons), we averaged the NMSE over the entire parameter space of (*a*,*ρ*) and obtained 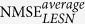. This procedure is iterated 50 times with the use of different LESNs for each, and the averaged 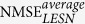 is obtained and used for comparisons. The second criteria is to compare with the performance of the optimal LESN in terms of 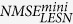, which was also defined in the main text. During the process of analyzing the entire parameter space of (*a*,*ρ*) as explained above, we determine the minimum NMSE. This is defined as an 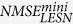 for an LESN, and the averaged 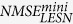 is calculated with the use of 50 different LESNs and is then used for the analyses. Note that even if the parameters of *a* and *ρ* are exactly the same, the performance of LESN differs due to the arbitrariness of the randomly assigned 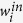 and *w*_*ij*_ for each LESN.

Supplementary Figure S4(a) shows the averaged ratio of (*a*,*ρ*)-parameters against the entire parameter space that shows lower values of NMSE than NMSE_*system*_ (the performance is better than that of our system) for each task with each *T* setting. We can see that in the NARMA2 task, when *T* = 100, the averaged ratio is higher than around 0.7, and then when *T* = 150, the averaged ratio suddenly drops to less than 0.4. According to the increase of the parameter *T* from 150, the averaged ratio also grows step by step. In particular, when *T* = 150, 200, 250, and 300, the averaged ratio tends to be less than 0.5 or around 0.5, which means that LESN is worse than our system in more than or almost half of the parameters in the entire parameter space. This result also suggests that for this task in these *T* settings, if we randomly assign the parameters *a* and *ρ* for the reservoir of LESN, it is likely to have an LESN that has a similar or worse performance than that of our system. The same conclusion can be made for NARMA5, NARMA10, NARMA15, and NARMA20 tasks for all the settings of *T* (Supplementary Fig. S4(a)). Supplementary Figure S4(b) shows a typical example of the NMSE for a single LESN over the entire (*a*,*ρ*)-space. We can see that the color plots clearly demonstrate the tendency observed in Supplementary Fig. S4(a).

### Statistical comparisons of the task performance with LR and LESN

To statistically compare the task performance of a simple LR system defined in the main text and LESN with that of our system, we used significant tests based on the respective NMSEs for each experimental condition. To compare NMSE_*system*_ and NMSE_*LR*_, we used a standard paired t-test for each experimental condition and checked the significant difference. Because the comparisons of 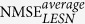 and 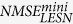 with NMSE_*system*_ are unpaired samples, we first employed a standard f-test to investigate whether we can assume equal variances between samples. When we could assume equal variances, we utilized Student’s t-test to check the significant difference; if not, we used Welch’s t-test. Supplementary Table S1 summarizes all the information, including the results of the t-test.

## Additional Information

**How to cite this article**: Nakajima, K. *et al.* Information processing via physical soft body. *Sci. Rep.* **5**, 10487; doi: 10.1038/srep10487 (2015).

## Supplementary Material

Supplementary Information

Supplementary Information
