# Supplementary material for: Information processing via physical soft body
Source: Sci Rep. 2015 May 27;5:10487. doi: 10.1038/srep10487 (PMC4444959; doi:10.1038/srep10487)
Supplement: Supplementary Information [file srep10487-s1.pdf]

Information processing via physical soft body

Supplementary Information

Kohei Nakajima[1*], Helmut Hauser[2], Tao Li[3], Rolf Pfeifer[4,5]

[1] The Hakubi Center for Advanced Research & Graduate School of Informatics, Kyoto University, 606-8501 Kyoto, Japan.
[2] Department of Engineering Mathematics, University of Bristol, Bristol BS8 1UB, United Kingdom.
[3] Department of Engineering and Information Technology, Bern University of Applied Sciences, 2501 Biel, Switzerland.
[4] Institute for Academic Initiatives, Osaka University, Osaka 560-8531, Japan.
[5] Department of Automation, Shanghai Jiao Tong University, Shanghai 200240, China.

[*] To whom correspondence should be addressed; email: k_nakajima@acs.i.kyoto-u.ac.jp

Supplementary Table

Supplementary Table S1: The averaged NMSE$_{system}$, NMSE$_{LR}$, NMSE$^{mini}_{LESN}$, and NMSE$^{average}_{LESN}$ are shown. For NMSE$_{LR}$, NMSE$^{mini}_{LESN}$, and NMSE$^{average}_{LESN}$, the results of significant tests in terms of p-value comparing with NMSE$_{system}$ are also depicted for each experimental condition. Note that "n.s." represents "not significant."

Task	T	NMSE$_{system}$	NMSE$_{LR}$		NMSE$^{mini}_{LESN}$		NMSE$^{average}_{LESN}$	
		mean ± SD	mean ± SD	p-value	mean ± SD	p-value	mean ± SD	p-value
NARMA2	100	1.36±0.07 ($\times 10^{-5}$)	1.89±0.01 ($\times 10^{-5}$)	$p < 0.01$	2.30±2.35 ($\times 10^{-6}$)	$p < 0.01$	0.94±0.20 ($\times 10^{-5}$)	$p < 0.01$
	150	1.20±0.02 ($\times 10^{-5}$)	2.07±0.03 ($\times 10^{-5}$)	$p < 0.01$	1.99±2.72 ($\times 10^{-6}$)	$p < 0.01$	1.12±0.30 ($\times 10^{-5}$)	n.s.
	200	1.11±0.16 ($\times 10^{-5}$)	2.15±0.05 ($\times 10^{-5}$)	$p < 0.01$	1.59±1.81 ($\times 10^{-6}$)	$p < 0.01$	1.18±0.32 ($\times 10^{-5}$)	n.s.
	250	1.41±0.06 ($\times 10^{-5}$)	2.24±0.05 ($\times 10^{-5}$)	$p < 0.01$	0.86±1.02 ($\times 10^{-6}$)	$p < 0.01$	1.13±0.25 ($\times 10^{-5}$)	$p < 0.01$
	300	1.73±0.12 ($\times 10^{-5}$)	2.30±0.06 ($\times 10^{-5}$)	$p < 0.01$	0.56±0.94 ($\times 10^{-6}$)	$p < 0.01$	1.08±0.33 ($\times 10^{-5}$)	$p < 0.01$
	350	1.91±0.10 ($\times 10^{-5}$)	2.37±0.05 ($\times 10^{-5}$)	$p < 0.01$	0.72±1.33 ($\times 10^{-6}$)	$p < 0.01$	1.01±0.32 ($\times 10^{-5}$)	$p < 0.01$
	400	1.96±0.07 ($\times 10^{-5}$)	2.39±0.07 ($\times 10^{-5}$)	$p < 0.01$	0.36±0.50 ($\times 10^{-6}$)	$p < 0.01$	0.90±0.28 ($\times 10^{-5}$)	$p < 0.01$
NARMA5	100	1.50±0.04 ($\times 10^{-3}$)	3.10±0.01 ($\times 10^{-3}$)	$p < 0.01$	0.53±0.76 ($\times 10^{-3}$)	$p < 0.01$	1.72±0.90 ($\times 10^{-3}$)	n.s.
	150	3.14±0.10 ($\times 10^{-3}$)	6.69±0.07 ($\times 10^{-3}$)	$p < 0.01$	0.57±1.25 ($\times 10^{-3}$)	$p < 0.01$	2.91±2.13 ($\times 10^{-3}$)	n.s.
	200	3.57±0.25 ($\times 10^{-3}$)	8.88±0.14 ($\times 10^{-3}$)	$p < 0.01$	0.54±1.60 ($\times 10^{-3}$)	$p < 0.01$	3.40±2.55 ($\times 10^{-3}$)	n.s.
	250	3.54±0.70 ($\times 10^{-3}$)	1.02±1.75 ($\times 10^{-4}$)	$p < 0.01$	0.50±1.66 ($\times 10^{-3}$)	$p < 0.01$	3.94±2.82 ($\times 10^{-3}$)	n.s.
	300	4.06±0.90 ($\times 10^{-3}$)	1.10±2.01 ($\times 10^{-4}$)	$p < 0.01$	0.65±2.15 ($\times 10^{-3}$)	$p < 0.01$	4.34±3.01 ($\times 10^{-3}$)	n.s.
	350	2.90±0.55 ($\times 10^{-3}$)	1.16±1.61 ($\times 10^{-4}$)	$p < 0.01$	0.40±1.73 ($\times 10^{-3}$)	$p < 0.01$	4.96±3.06 ($\times 10^{-3}$)	$p < 0.01$
	400	2.54±0.38 ($\times 10^{-3}$)	1.19±2.65 ($\times 10^{-4}$)	$p < 0.01$	0.92±2.60 ($\times 10^{-3}$)	$p < 0.01$	5.43±3.53 ($\times 10^{-3}$)	$p < 0.01$
NARMA10	100	1.97±0.02 ($\times 10^{-3}$)	2.20±0.01 ($\times 10^{-3}$)	$p < 0.01$	0.84±0.46 ($\times 10^{-3}$)	$p < 0.01$	1.75±0.31 ($\times 10^{-3}$)	$p < 0.01$
	150	1.09±0.01 ($\times 10^{-3}$)	1.33±0.01 ($\times 10^{-3}$)	$p < 0.01$	0.39±0.25 ($\times 10^{-3}$)	$p < 0.01$	0.98±0.25 ($\times 10^{-3}$)	$p < 0.01$
	200	1.68±0.16 ($\times 10^{-3}$)	2.76±0.04 ($\times 10^{-3}$)	$p < 0.01$	0.37±0.45 ($\times 10^{-3}$)	$p < 0.01$	1.46±0.74 ($\times 10^{-3}$)	n.s.
	250	2.61±0.46 ($\times 10^{-3}$)	4.84±0.08 ($\times 10^{-3}$)	$p < 0.01$	0.36±0.90 ($\times 10^{-3}$)	$p < 0.01$	2.16±1.18 ($\times 10^{-3}$)	$p < 0.05$
	300	3.27±0.59 ($\times 10^{-3}$)	6.81±0.13 ($\times 10^{-3}$)	$p < 0.01$	0.67±1.57 ($\times 10^{-3}$)	$p < 0.01$	3.12±2.16 ($\times 10^{-3}$)	n.s.
	350	2.79±0.44 ($\times 10^{-3}$)	8.56±0.13 ($\times 10^{-3}$)	$p < 0.01$	0.67±1.77 ($\times 10^{-3}$)	$p < 0.01$	3.82±2.78 ($\times 10^{-3}$)	$p < 0.05$
	400	2.97±0.65 ($\times 10^{-3}$)	9.86±0.23 ($\times 10^{-3}$)	$p < 0.01$	0.68±1.83 ($\times 10^{-3}$)	$p < 0.01$	4.34±2.97 ($\times 10^{-3}$)	$p < 0.01$
NARMA15	100	2.81±0.03 ($\times 10^{-3}$)	3.76±0.01 ($\times 10^{-3}$)	$p < 0.01$	1.15±0.66 ($\times 10^{-3}$)	$p < 0.01$	2.87±0.57 ($\times 10^{-3}$)	n.s.
	150	2.40±0.07 ($\times 10^{-3}$)	2.70±0.05 ($\times 10^{-3}$)	$p < 0.01$	0.93±0.56 ($\times 10^{-3}$)	$p < 0.01$	2.13±0.35 ($\times 10^{-3}$)	$p < 0.01$
	200	1.34±0.02 ($\times 10^{-3}$)	1.44±0.02 ($\times 10^{-3}$)	$p < 0.01$	0.43±0.29 ($\times 10^{-3}$)	$p < 0.01$	1.09±0.20 ($\times 10^{-3}$)	$p < 0.01$
	250	1.46±0.11 ($\times 10^{-3}$)	1.75±0.01 ($\times 10^{-3}$)	$p < 0.01$	0.36±0.32 ($\times 10^{-3}$)	$p < 0.01$	1.11±0.32 ($\times 10^{-3}$)	$p < 0.01$
	300	1.94±0.17 ($\times 10^{-3}$)	2.84±0.04 ($\times 10^{-3}$)	$p < 0.01$	0.34±0.56 ($\times 10^{-3}$)	$p < 0.01$	1.47±0.64 ($\times 10^{-3}$)	$p < 0.01$
	350	2.15±0.22 ($\times 10^{-3}$)	4.39±0.07 ($\times 10^{-3}$)	$p < 0.01$	0.32±0.64 ($\times 10^{-3}$)	$p < 0.01$	2.12±1.08 ($\times 10^{-3}$)	n.s.
	400	2.62±0.46 ($\times 10^{-3}$)	6.04±0.15 ($\times 10^{-3}$)	$p < 0.01$	0.61±1.41 ($\times 10^{-3}$)	$p < 0.01$	2.93±1.71 ($\times 10^{-3}$)	n.s.
NARMA20	100	1.66±0.01 ($\times 10^{-3}$)	1.73±0.01 ($\times 10^{-3}$)	$p < 0.01$	0.83±0.22 ($\times 10^{-3}$)	$p < 0.01$	1.51±0.12 ($\times 10^{-3}$)	$p < 0.01$
	150	3.69±0.08 ($\times 10^{-3}$)	4.26±0.09 ($\times 10^{-3}$)	$p < 0.01$	1.26±0.64 ($\times 10^{-3}$)	$p < 0.01$	3.34±0.51 ($\times 10^{-3}$)	$p < 0.01$
	200	2.55±0.08 ($\times 10^{-3}$)	2.95±0.08 ($\times 10^{-3}$)	$p < 0.01$	0.92±0.51 ($\times 10^{-3}$)	$p < 0.01$	2.41±0.30 ($\times 10^{-3}$)	$p < 0.01$
	250	1.77±0.05 ($\times 10^{-3}$)	1.84±0.04 ($\times 10^{-3}$)	$p < 0.01$	0.55±0.35 ($\times 10^{-3}$)	$p < 0.01$	1.48±0.18 ($\times 10^{-3}$)	$p < 0.01$
	300	1.60±0.06 ($\times 10^{-3}$)	1.81±0.02 ($\times 10^{-3}$)	$p < 0.01$	0.47±0.31 ($\times 10^{-3}$)	$p < 0.01$	1.37±0.22 ($\times 10^{-3}$)	$p < 0.01$
	350	1.81±0.11 ($\times 10^{-3}$)	2.36±0.02 ($\times 10^{-3}$)	$p < 0.01$	0.43±0.31 ($\times 10^{-3}$)	$p < 0.01$	1.55±0.40 ($\times 10^{-3}$)	$p < 0.01$
	400	2.18±0.19 ($\times 10^{-3}$)	3.35±0.06 ($\times 10^{-3}$)	$p < 0.01$	0.54±0.74 ($\times 10^{-3}$)	$p < 0.01$	2.00±0.77 ($\times 10^{-3}$)	n.s.

Supplementary Figures

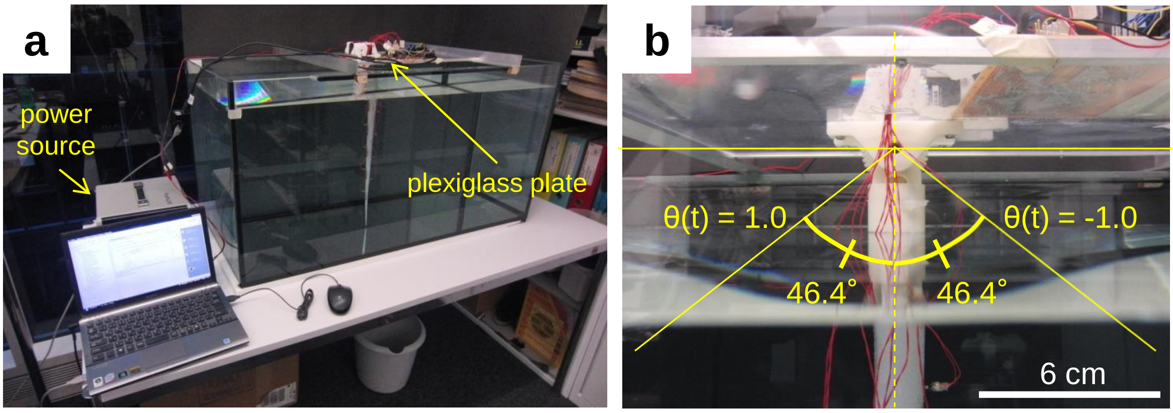

Supplementary Figure S1: Pictures showing the overall platform (a) and a close-up of the arm base (b). For (b), the maximum left ($\theta(t) = 1.0$) and right ($\theta(t) = -1.0$) base positions, which are 46.4 degrees, are depicted. See Methods section for details.

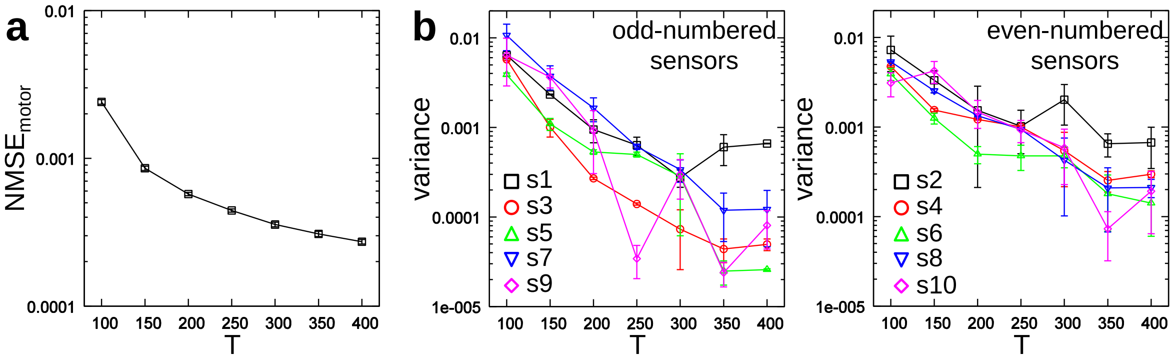

Supplementary Figure S2: The averaged NMSE$_{motor}$ between $m(t)$ and $\theta(t)$ (a) and the averaged variance for each sensory value according to the parameter T of the input among 20 experimental trials (b). For (b), left and right diagrams plot the odd-numbered sensors and even-numbered sensors, respectively. For all plots, the vertical axis is in a log scale, and the error bars show the standard deviations.

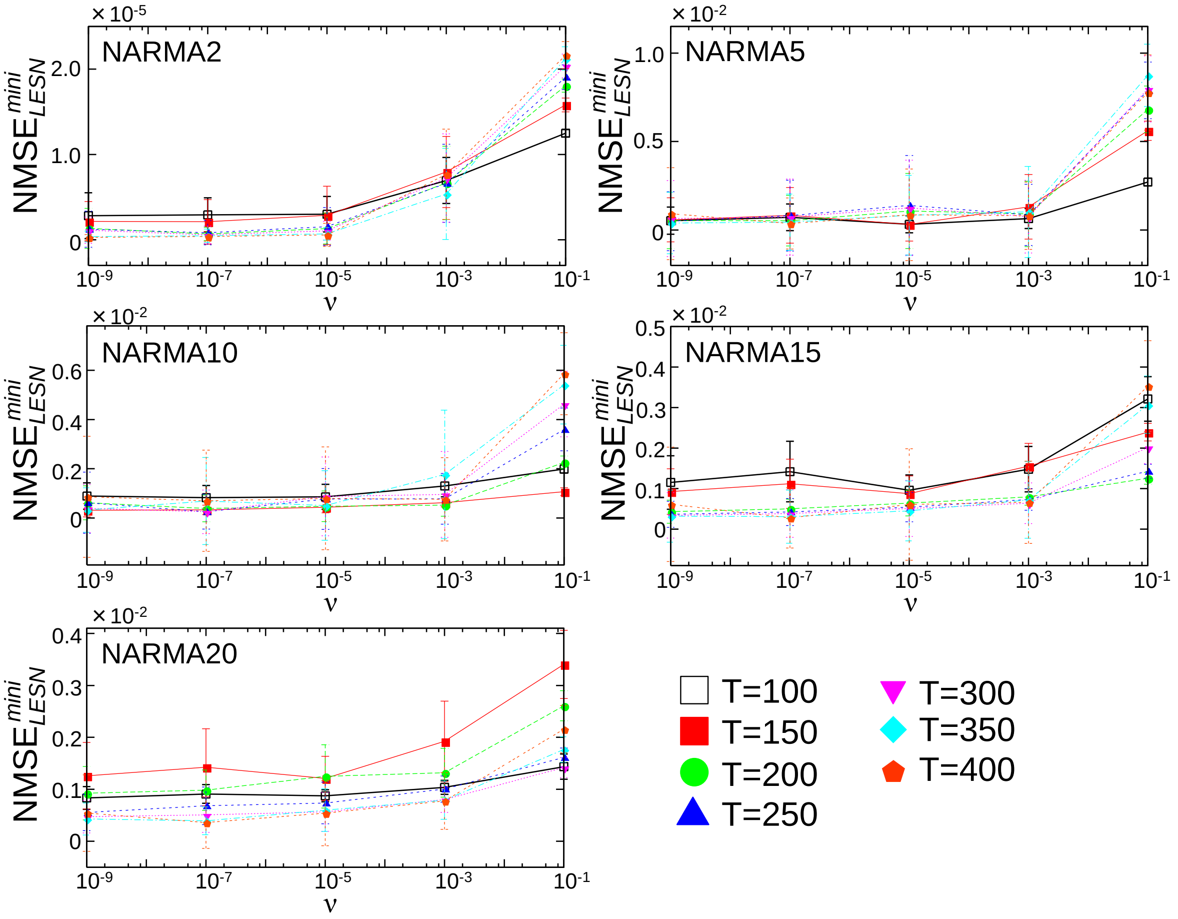

Supplementary Figure S3: Plots showing the averaged $\mathrm{NMSE}^{mini}_{LESN}$ according to each noise intensity ν for 5 NARMA tasks. For each plot, the cases from $T = 100$ to $T = 400$ are overlaid. The averaged $\mathrm{NMSE}^{mini}_{LESN}$ is calculated by using 30 different LESNs for each condition, and the error bars show the standard deviations for each plot.

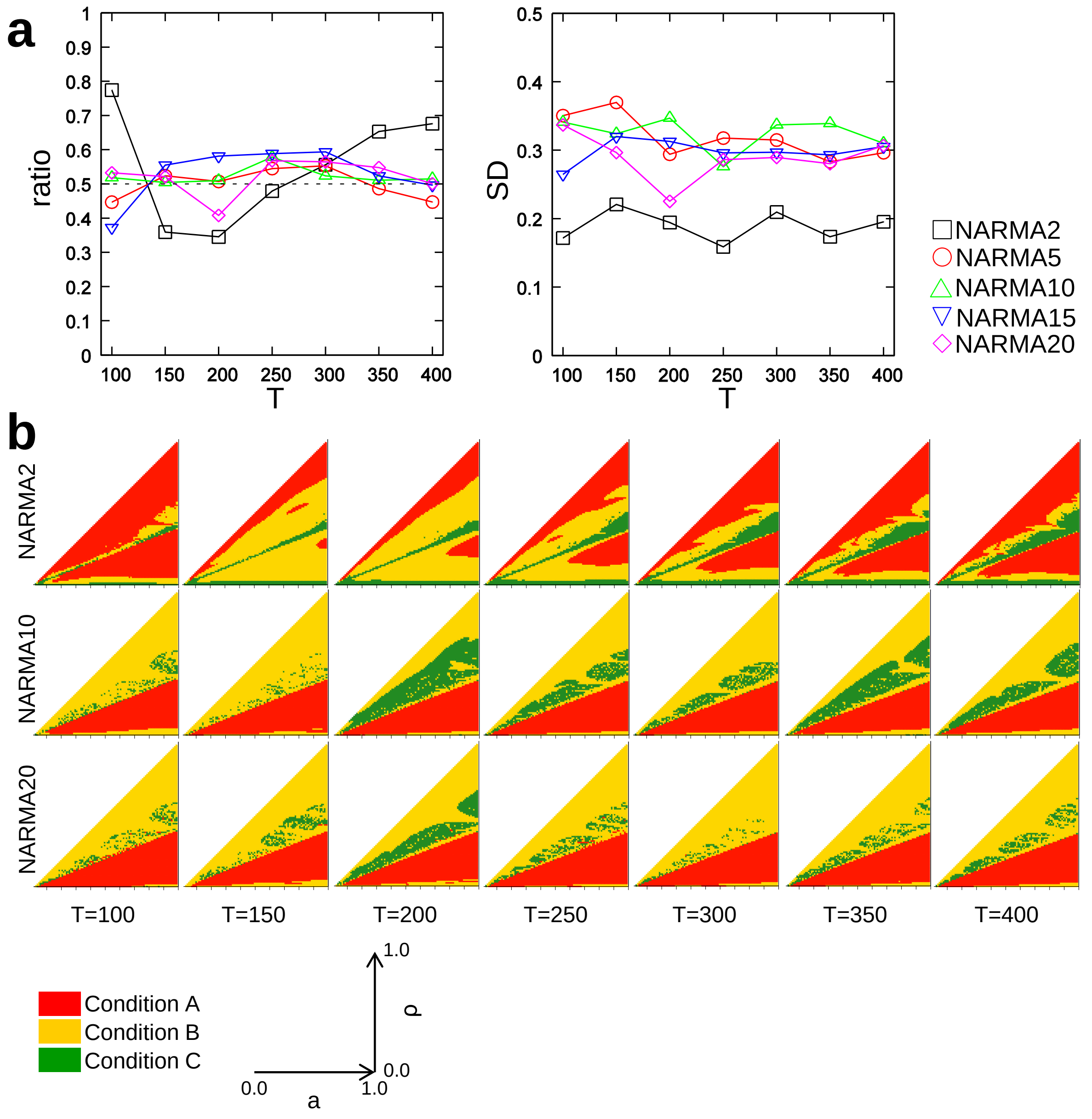

Supplementary Figure S4: The averaged ratio of NMSEs for the LESN that shows lower value than the NMSE$_{system}$ against the entire (a, ρ)-space (a), and a typical example of the NMSE for each parameter of a and ρ (b). For (a), the averaged ratios for NARMA tasks for each setting of T are overlaid in the left plot, and the corresponding standard deviations are plotted in the right for clarity. The plots show the averaged value over 50 different LESNs. For (b), NMSEs in the condition of NMSE $<$ NMSE$_{system}$ are classified as "Condition A," NMSEs in the condition of NMSE$_{system}$ $\leq$ NMSE $<$ NMSE$_{LR}$ are classified as "Condition B," and NMSEs in the condition of NMSE$_{LR}$ $\leq$ NMSE are classified as "Condition C" and colored differently in the plot. The plots for NARMA5 and NARMA15 tasks showed a tendency that was similar to NARMA10 and NARMA20 tasks.

Supplementary Video
(Caption)

Supplementary Video S1: A video showing the arm motion, the corresponding sensory responses, and the system performance for 5 NARMA tasks for each setting of T during the evaluation phase. The cases for $T = 100, 200, 300,$ and 400 are shown for example.
